# Cytomegalovirus immune evasion sets the functional avidity threshold for protection by CD8 T cells

**DOI:** 10.1007/s00430-022-00733-w

**Published:** 2022-04-01

**Authors:** Sara Hamdan, Matthias J. Reddehase, Rafaela Holtappels

**Affiliations:** Institute for Virology and Research Center for Immunotherapy (FZI), University Medical Center of the Johannes Gutenberg-University Mainz, Obere Zahlbacher Strasse 67, Hochhaus Am Augustusplatz, 55131 Mainz, Germany

**Keywords:** Allogeneic hematopoietic cell transplantation (allo-HCT), Adoptive cell transfer, Antigen presentation, Antiviral protection, Avidity, CD8 T cells, Cytomegalovirus, Immune evasion, Immunoevasin, Immunotherapy, T effector cells (TEC)

## Abstract

Conflicting hallmarks are attributed to cytomegalovirus (CMV) infections. CMVs are viewed as being master tacticians in “immune evasion” by subverting essentially all pathways of innate and adaptive immunity. On the other hand, CMV disease is undeniably restricted to the immunologically immature or immunocompromised host, whereas an intact immune system prevents virus spread, cytopathogenic tissue infection, and thus pathological organ manifestations. Therefore, the popular term “immune evasion” is apparently incongruous with the control of CMV infections in the immunocompetent human host as well as in experimental non-human primate and rodent models. Here, we review recent work from the mouse model that resolves this obvious discrepancy for the example of the virus-specific CD8 T-cell response. Immune evasion proteins encoded by murine CMV (mCMV) interfere with the cell surface trafficking of antigenic peptide-loaded MHC class-I (pMHC-I) complexes and thereby reduce their numbers available for interaction with T-cell receptors of CD8 T cells; but this inhibition is incomplete. As a consequence, while CD8 T cells with low interaction avidity fail to receive sufficient signaling for triggering their antiviral effector function in the presence of immune evasion proteins in infected cells, a few pMHC-I complexes that escape to the cell surface are sufficient for sensitizing high-avidity CD8 T cells. It is thus proposed that the function of immune evasion proteins is to raise the avidity threshold for activation, so that in the net result, only high-avidity cells can protect. An example showing that immune evasion proteins can make the difference between life and death is the lacking control of infection in a mouse model of MHC-I histoincompatible hematopoietic cell transplantation (allogeneic-HCT). In this model, only low-avidity CD8 T cells become reconstituted by HCT and almost all infected HCT recipients die of multiple-organ CMV disease when immune evasion proteins are expressed. In contrast, lowering the avidity threshold for antigen recognition by deletion of immune evasion proteins allowed control of infection and rescued from death.

## Introduction

Cytomegaloviruses (CMVs) belong to the β-subfamily of the herpes virus family and have co-evolved with their mammalian hosts for estimated 350 million years (for overviews, see [1, 2]). This co-evolution has led to perfect adaptation of any CMV species to its respective host. This accounts for host-species specificity of virus replication [3–5], balance of immune control and immune evasion [6], as well as the establishment and maintenance of latent infection, referred to as “latency” (for more recent reviews, see [7–10]). As a consequence, the clinically relevant human CMV (hCMV) cannot be studied in experimental animal models, with the exception of specific but limited questions that can be addressed in humanized mouse models with human tissue implants [11, 12]. It is a matter of course that ethical concerns limit the clinical investigation of CMV pathogenesis and immune control to natural primary infection and virus reactivation from latency in clinical settings, whereas experimental genetic manipulation of virus and/or host for scientific purposes is out of the question. Although non-human primate models are closest to human infection, they nonetheless differ in important aspects of CMV pathogenesis and immune control, are limited with respect to manipulation of host genetics, and may also raise ethical concerns [13–15]. Despite pronounced genetic differences in both virus and host between human infection and experimental animal models, convergent evolution resulted in molecularly different but functionally analogous mechanisms of virus–host interplay. As we have reviewed recently, the mouse model based on infection with murine CMV (mCMV) has identified principles of pathogenesis and immune control that have proven valid also for hCMV [16]. Specifically, clinical immunotherapy of hCMV infection in immunocompromised recipients of hematopoietic cell transplantation (HCT) by transfer of viral epitope-specific CD8 T cells [17–20] has been pioneered by preclinical data from the mouse model of experimental HCT and mCMV infection (reviewed in [21–23]).

A common feature shared by all CMVs is the rapid control of primary infection of the immunocompetent host by innate and adaptive immune responses. These prevent an extensive viral spread and overt organ manifestations caused by cytopathogenic tissue infection, but fail to clear viral genomes and thus result in the establishment of latency. Latency is maintained by continuous immune surveillance that prevents productive reactivation [24, 25]. CMV organ disease can develop after primary infection of an immunologically immature or immunodeficient host or by recurrent infection after productive reactivation of latent virus under immunocompromising conditions that abrogate immune surveillance. Accordingly, the risk of CMV disease leading to birth defects results from infection of the fetus after primary or recurrent infection of pregnant women (for overviews, see [26, 27]) and virus reactivation from latency is a feared complication in iatrogenically immunocompromised transplant recipients.

Specifically, in solid organ transplantation (SOT), immunosuppressive prophylaxis or therapy of a host-versus-graft (HvG) response to prevent immune-mediated graft rejection bears a risk of virus-mediated graft loss due to latent virus reactivation. This mostly occurs in the transplanted organ from a CMV-latent donor rather than in the CMV-latent recipient’s organs, as indicated by the recurrence of donor-type CMV ([28–31], discussed in [9]).

In HCT, transient immunodeficiency due to hemato-ablative therapy of the primary disease, such as leukemia, favors reactivation of latent CMV in a “window of risk” between HCT and successful immune system reconstitution. An often lethal interstitial pneumonia (CMV-IP) is the most deleterious clinical manifestation of recurrent CMV infection in HCT recipients (for more recent reviews, see [32–34]). Dependent on CMV status, virus reactivation can originate in the hematopoietic cell transplant, or in organs of the recipient, or in both. Notably, in HCT, the recurrent virus is more frequently of recipient-type, which indicates that hematopoietic stem and/or progenitor cells are not the predominant source of latent CMV ([31], reviewed in [9]). An additional risk of CMV reactivation and disease is posed in allogeneic-HCT (allo-HCT) by a graft-versus-host (GvH) reaction against mismatches in major and/or minor histocompatibility antigens, that is in MHC/HLA and/or in minor-H antigens (mHAg), respectively. On top of this, immunosuppressive prophylaxis or therapy of GvH disease (GvHD) further promotes lethal CMV disease by preventing the reconstitution of protective, antiviral CD8 T cells (for more recent reviews, see [35, 36]).

All in all, the lesson from clinical reality tells us that CMV disease is a typical “disease of the immunocompromised host”. This contrasts fundamentally with numerous basic science reports on CMV “immune evasion” by encoding proteins that subvert essentially all pathways of intrinsic host cell defense as well as of innate and adaptive immune responses (for more recent reviews, see [37–40]). Accordingly, clinicians and basic scientists talk about CMV in different languages.

Here, we review recent work showing that in the case of CMV control by CD8 T cells, the key to resolve the seeming contradiction is CD8 T-cell avidity in antigen recognition.

## Selection of high-avidity and low-avidity CD8 T effector cells

Studies on antigen presentation by CMV-infected cells to CD8 T cells were usually performed in cell culture using lines of CD8 T effector cells (TEC) as probes for detection. TEC lines were propagated with optimized doses of an antigenic peptide of interest with the aim to stimulate and expand most if not all cells that express T-cell receptors (TCR) specific for a corresponding peptide-MHC class-I (pMHC-I) complex [41, 42]. TCRs differ in their structural avidities of binding to monomeric pMHC-I complexes in a Gaussian-like distribution ranging from low to high avidities, measured as TCR-ligand *k*_off_ rates [43]. In addition to TCR structural avidity, TCR cell surface density and accessory molecules at the TEC-target cell synapse contribute cooperatively to the functional avidity of the interaction by receptor clustering [44]. Therefore, it is predictable that CD8 T cells of high functional avidity are of superior sensitivity for detecting limited numbers of pMHC-I complexes at the surface of infected cells.

**Fig. 1:**
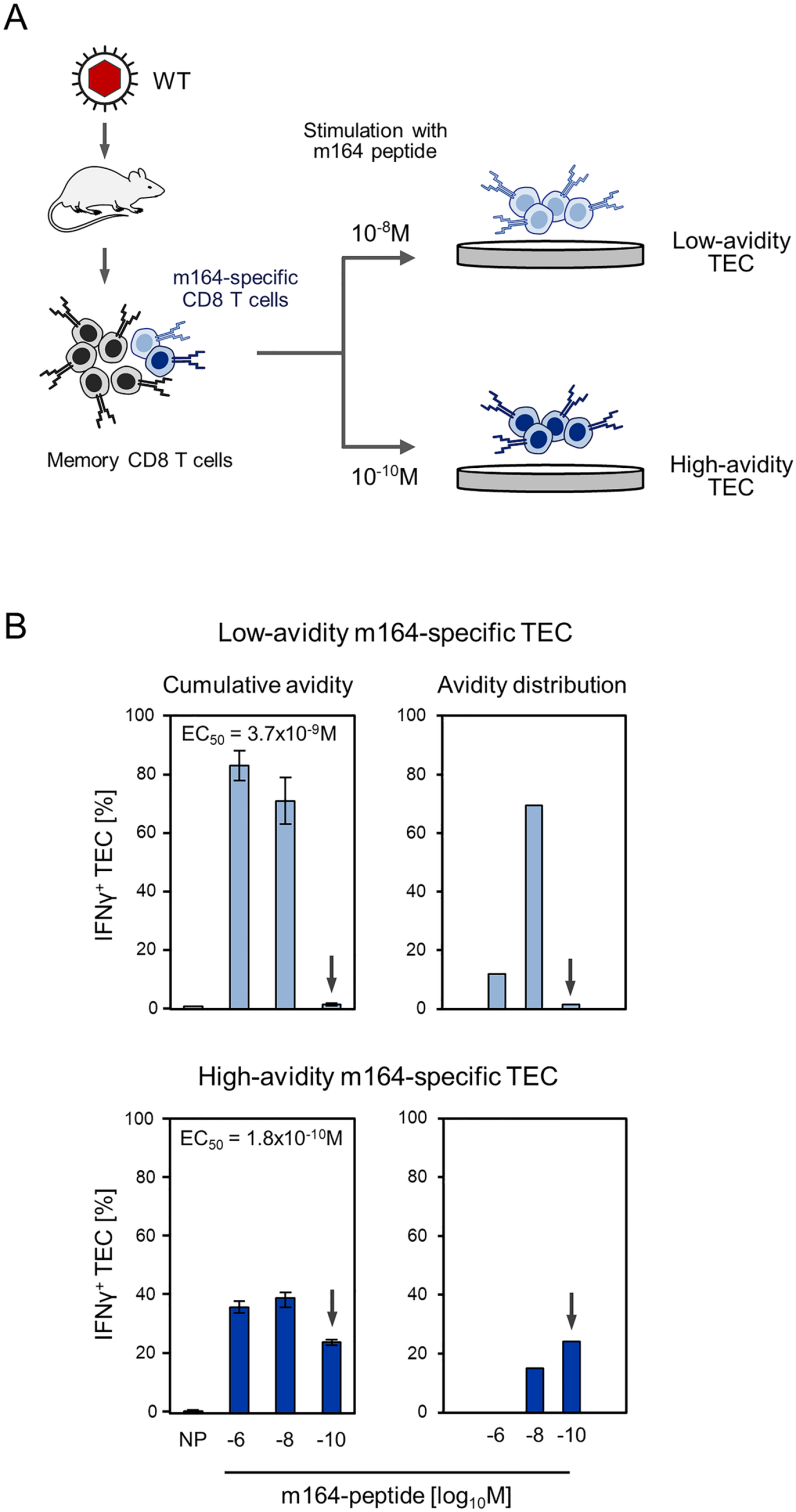
Selection of low-avidity and high-avidity TEC lines. **A** Scheme of the approach. The red-filled capsid in the wild-type (WT) virus symbol indicates the presence of vRAP-encoding genes. Symbols in light blue and dark blue color indicate low avidity and high avidity, respectively. *TEC* T effector cells. **B** Frequencies of TEC responding to presented antigenic peptide with IFNγ secretion in an ELISpot assay. (Left panels) cumulative avidity distributions. Bars represent frequencies of TEC (error bars: 95% confidence intervals) responding in the assay to stimulation by embryonic fibroblasts exogenously loaded with synthetic antigenic peptide m164 at the graded molar concentrations indicated. *NP* no peptide. EC_50_, effective concentration of antigenic peptide that leads to the half-maximal response. (Right panels) Gaussian-like avidity distributions. Bars represent the calculated increase in responding TEC frequencies between lower peptide concentrations and the peptide concentration indicated. These values quantitate TEC with an avidity defined by the corresponding peptide concentration. Arrows mark the detection threshold for the low-avidity TEC line. Reproduced in modified form and with additional calculations of EC_50_ values from an experiment published in reference [45]

As tools for studying the impact of functional avidity on the recognition of target cells, low-avidity and high-avidity TEC lines were selected from polyclonal memory CD8 T cells by stimulation and propagation in cell culture with high (10^–8^ M) and low (10^–10^ M) concentrations of antigenic peptide, respectively (Fig. 1A, [22]). The antigenic peptide m164 (amino acid sequence AGPPRYSRI) of mCMV, which is presented by the MHC-I molecule D^d^, was chosen based on the previous finding that a broad range of TCR avidities in the memory cell population facilitates the selection of m164-specific TEC lines that differ in the target cell peptide loading concentration required for half-maximal cytolytic effector function [41]. In the present example, the effector function read-out was IFNγ secretion, and IFNγ^+^ TEC responding to target cells were quantitated for graded peptide concentrations used for exogenous target cell loading (Fig. 1B, [45]). The 50% effective concentration (EC_50_) values differed by a factor of ca. 20, namely 3.7 × 10^–9^ M for the low-avidity TEC line and 1.8 × 10^–10^ M for the high-avidity TEC line. Based on this, only the high-avidity TEC line included cells able to recognize target cells exogenously loaded with 10^–10^ M of synthetic m164 peptide, whereas most cells of the low-avidity TEC line were sensitized by 10^–8^ M for IFNγ secretion as effector function.

## Only high-avidity TEC recognize infected cells that express immune evasion proteins

**Fig. 2:**
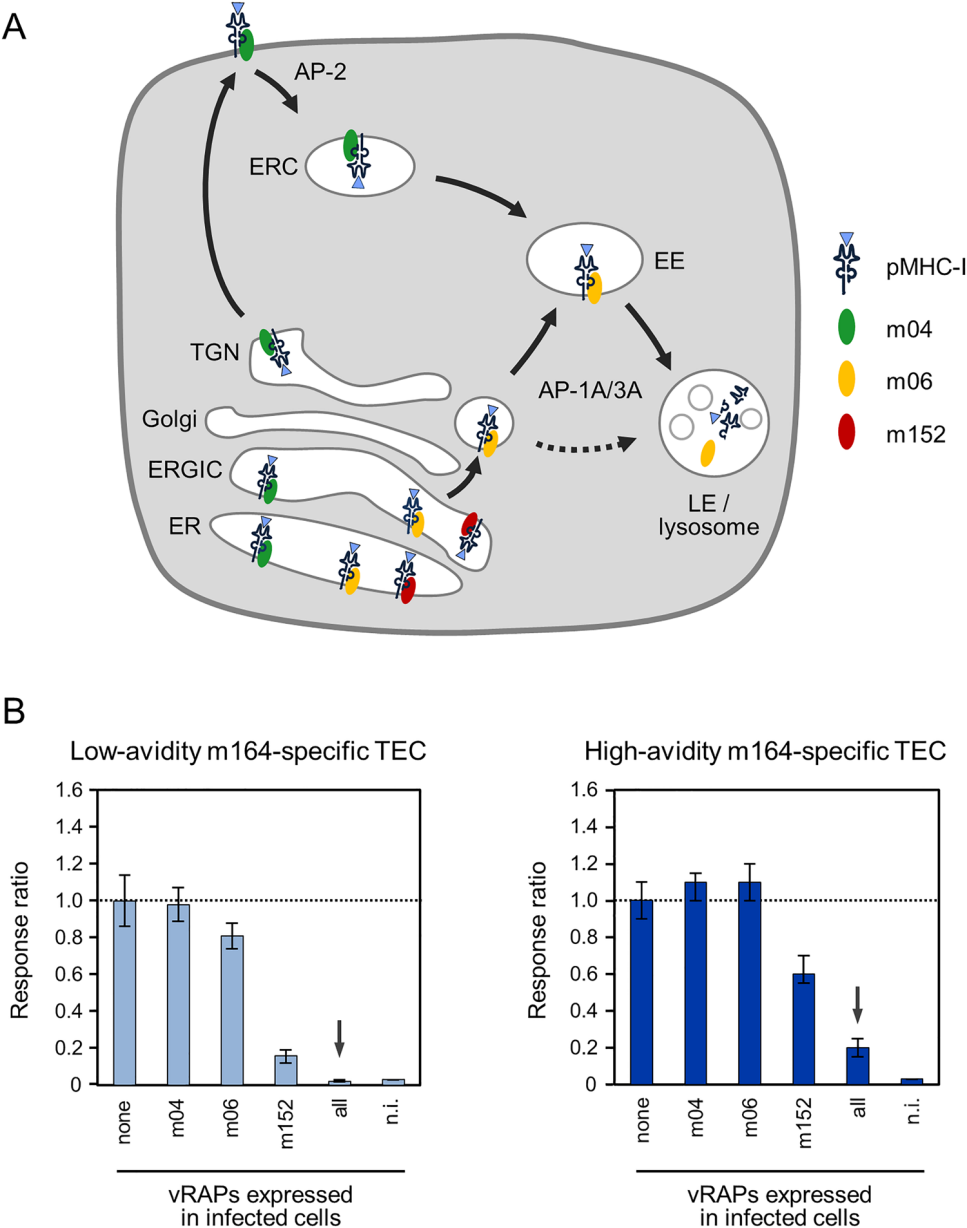
High avidity overcomes immune evasion. **A** Current view of the concerted function of vRAPs. *ER* endoplasmic reticulum, *ERGIC* ER-Golgi Intermediate Compartment, *TGN* *Trans*-Golgi Network, *ERC* Endosomal Recycling Compartment, *EE* Early Endosome, *LE* Late Endosome, *AP* Adapter Protein. For more detailed explanation, see the body of the text and reference [40]. **B** Avidity of TEC defines the sensitivity of detecting presented antigen. Bars represent the responses of TEC of the low-avidity and high-avidity TEC lines defined in Fig. 1, normalized to the response to target/stimulator cells not expressing vRAPs after infection with the triple deletion mutant mCMV-ΔvRAP. Error bars represent the 95% confidence intervals. The IFNγ ELISpot assay was performed with target/stimulator cells expressing the indicated vRAPs after infection with the corresponding combinatorial vRAP gene deletion mutants of mCMV. All, infection with the parental virus expressing all three vRAPs; n.i., uninfected cells. Reproduced, with modification, from reference [45]

In infected cells, pMHC-I complexes are formed in the ER after endogenous, mostly proteasomal processing of viral proteins, peptide translocation into the ER, and peptide loading on nascent MHC-I molecules. Cell surface display of pMHC-I complexes for recognition by CD8 T cells is regulated in mCMV-infected cells by synergistic and antagonistic action of proteins (viral regulators of antigen presentation, vRAPs) that dictate the intracellular trafficking of recently folded pMHC-I complexes from the ER to the cell surface or intracellular compartments [46] by hijacking intracellular cargo sorting pathways (comprehensively reviewed in [40]). In essence, the functionally predominant “immune evasion” protein m152/gp40 of mCMV [47–49] traps pMHC-I complexes in an ER *cis*-Golgi intermediate compartment (ERGIC), whereas the *m02* gene family members m04/gp34 and m06/gp48 compete for pMHC-I cargo by binding in the ER and antagonize each other by guiding pMHC-I to the cell surface or by entering the endosomal-lysosomal pathway for disposal, respectively (Fig. 2A, [40]). In the net effect, the concerted action of these three vRAPs significantly limits the cell surface presentation of pMHC-I complexes.

For evaluating the impact of vRAPs, individually or all three in concert, target cells were infected with deletion mutants of mCMV expressing no vRAP or single vRAPs, or were infected with the parental virus expressing the full set of vRAPs (Fig. 2B, [45]). When tested with the low-avidity m164-specific TEC line, the number of TEC sensitized by cell surface pMHC-I (m164-D^d^ in the specific cells) was reduced by expression of m06 and even more reduced by m152. No cells in the TEC population recognized target cells expressing all three vRAPs. This finding bears the risk of falsely concluding that vRAPs would completely prevent antigen presentation [50]. When the same set of target cells was tested with the high-avidity m164-specific TEC line, no reduction in the number of responding TEC was observed for the expression of m06 and, compared to the low-avidity TEC line, the reduction by m152 expression was less. Importantly, a fraction of the cells in this high-avidity TEC line recognized target cells despite the expression of all three vRAPs. This finding proved that vRAPs do not completely prevent antigen presentation and that functional avidity of TEC is decisive for the recognition of infected cells.

## Immune evasion proteins prevent protection by low-avidity but not by high-avidity TEC

It almost goes without saying that high-avidity TEC are sensitized more efficiently than low-avidity TEC due to a prolonged interaction time between a TCR and a presented pMHC-I complex, that is a lower *k*_off_ rate, and due to a lower number of such interactions required at the immunological synapse for triggering effector function. It was thus highly predictable that for an immunotherapy of infection by adoptive cell transfer, high-avidity TEC are superior over low-avidity TEC in recognizing cells with limited antigen presentation and thus in protection in vivo against infection and virus spread in host tissues [22, 43, 51].

**Fig. 3:**
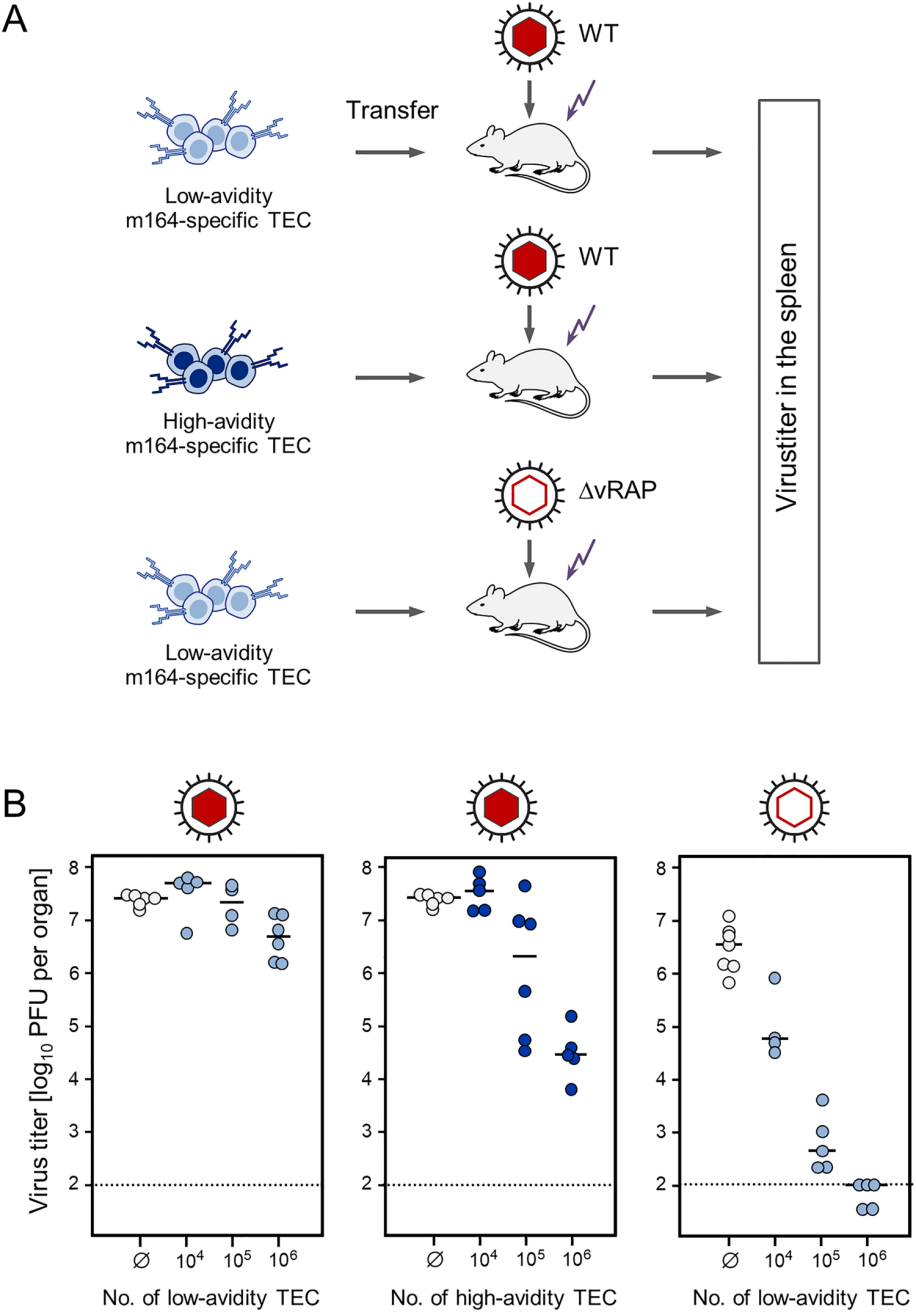
Impact of functional avidity of TEC on in vivo antiviral protection. **A** Scheme of the adoptive cell transfer approach. Symbols in light blue and dark blue color indicate low avidity and high avidity, respectively. TEC specific for the presented antigenic peptide m164 were transferred into BALB/c recipient mice that were immunocompromised by total-body γ-irradiation (flash symbol) and infected with either wild-type (WT) mCMV encoding vRAPs (symbolized by a red-filled capsid) or with the vRAP gene deletion mutant mCMV-ΔvRAP (symbolized by a red-rimmed empty capsid). **B** Virus titers in the spleen of the adoptive transfer recipients, measured as plaque-forming units (PFU), were determined on day 11 after transfer of TEC in numbers indicated. Dot symbols represent mice tested individually. Median values are marked. Light blue and dark blue colors indicate low avidity and high avidity, respectively. Ø, no cell transfer (empty circles). The dotted lines represent the detection limit of the infectivity assay. Reproduced in modified arrangement based on an experiment published in reference [22]

Here, we discuss a link between TEC avidity and the phenomenon of immune evasion in CMV infections. As shown above exemplarily in the mCMV model, the concerted action of vRAPs largely reduces antigen presentation, so that only TEC with high avidity can detect trace amounts of pMHC-I complexes at the cell surface. Accordingly, in an immunotherapy approach by adoptive cell transfer (Fig. 3, [22]), even high numbers of low-avidity TEC failed to control infection of immunocompromised recipient mice after infection with wild-type (WT) mCMV. In contrast, under otherwise identical conditions, high-avidity TEC controlled the infection in a dose-dependent manner. Most instructive with respect to explaining the difference is the finding of very efficient protection even by low-avidity TEC, provided that recipients were infected with a vRAP gene deletion mutant mCMV-ΔvRAP.

This experiment unequivocally identified reduction of target cell antigen presentation by vRAPs, rather than a T-cell intrinsic signaling deficiency, as the cause of missing protection by low-avidity TEC.

## CD8 T-cell avidity makes the difference between survival and death from CMV disease in allo-HCT

**Fig. 4:**
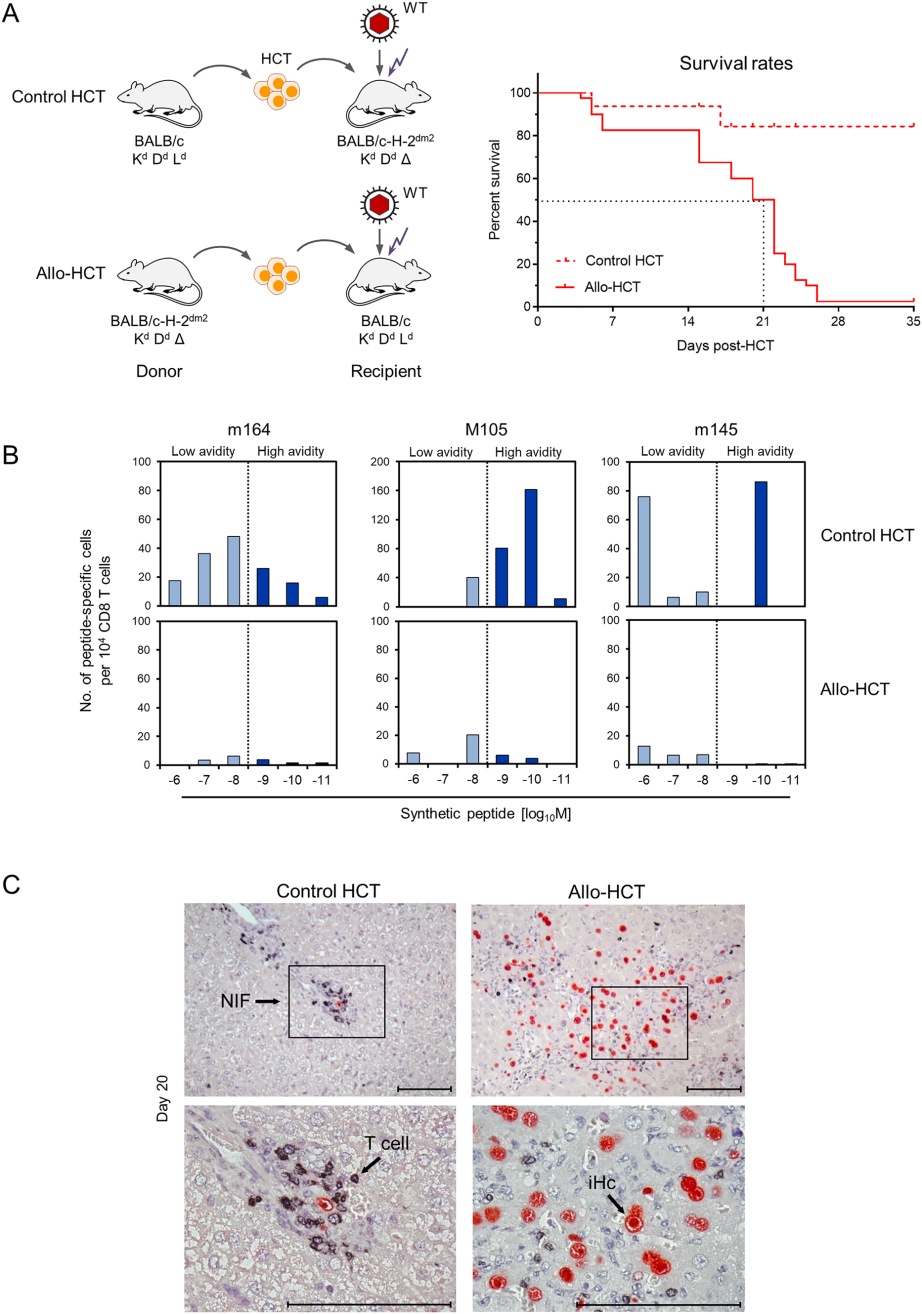
Lethal CMV disease after allo-HCT corresponds to inefficient reconstitution of high-avidity CD8 T cells. **A** Experimental HCT protocols and survival rates of the infected HCT recipients. HCT donors and recipients were chosen to differ in the expression of the MHC-I antigen L^d^ by either expressing it or lacking it (symbol Δ). Control HCT, no potential for a GvH response, because the target histocompatibility antigen L^d^ is not expressed in the recipients. Allo-HCT, potential for a GvH response, because the target histocompatibility antigen L^d^ is expressed in the recipients. **B** Gaussian-like avidity distributions (see Fig. 1B for explanation) of recipients’ liver-infiltrating CD8 T cells (day 20 post-HCT) specific for the viral antigenic peptides indicated. **C** Corresponding liver histopathology. (Control HCT) Liver infection is controlled and eventually resolved by liver-infiltrating T cells. (Allo-HCT), lack of liver-infiltrating T cells associated with uncontrolled, disseminated liver infection. Infected liver cells, which are predominantly hepatocytes (iHc), are identified by red staining of the intranuclear viral protein IE1. Tissue-infiltrating T cells are identified by black staining of the CD3ε molecule. Frames in the upper images demarcate the tissue regions that are resolved to greater detail in the lower images. *NIF* nodular inflammatory focus consisting of T cells aggregating around infected cells. Bar markers, 100 μm. Modified and presented in a new arrangement, based on references [36, 52]

A critical link between CD8 T-cell avidity and limited antigen presentation caused by CMV-encoded vRAPs has recently been identified in mouse models of MHC-I and of minor histocompatibility antigen-mismatched allo-HCTs ([52, 53], reviewed in [36]). It is long-established clinical experience that lethality in CMV-infected HCT recipients is associated with disparity in histocompatibility antigens between HCT donor and recipient, and it was proposed that this results from a fatal pathogenetic interplay between CMV infection and a graft-versus-host (GvH) reaction, enhancing each other (for reviews, see [35, 36]). The two mouse models of allo-HCT, however, concordantly identified an uncontrolled virus spread and extensive viral histopathology as the cause of death in the absence of GvH-reactive cells and the consequent absence of a histopathology characteristic of GvH disease (GvHD). Instead, a quantitative failure in the reconstitution of protective high-avidity CD8 T cells caused by histoincompatibility in itself was found to account for lethal CMV organ disease. In contrast, in control HCT models designed to avoid recognition of histocompatibility antigens in the HCT recipients, high-avidity CD8 T cells were reconstituted, resolved tissue infection, and prevented lethality (Fig. 4, [52, 53]).

**Fig. 5:**
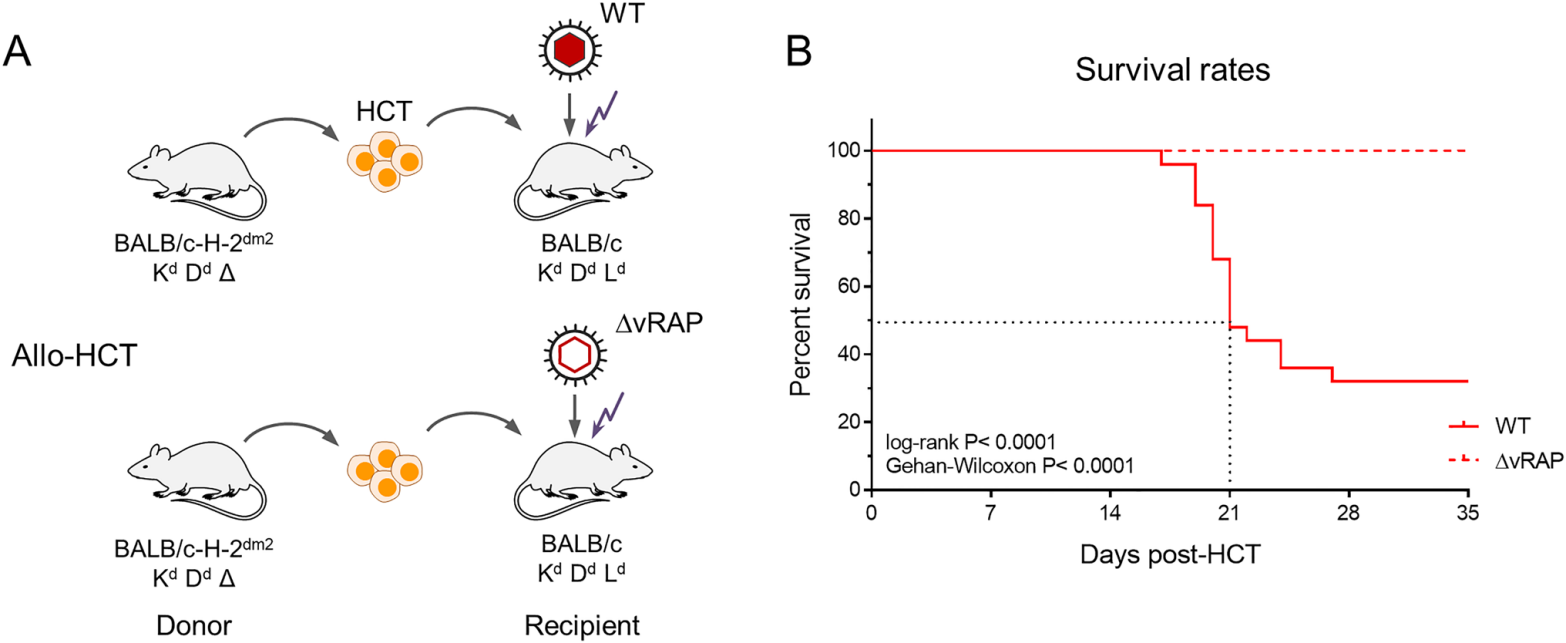
Deletion of vRAPs prevents lethality from CMV infection after allo-HCT. **A** Experimental allo-HCT protocols (see also Fig. 4). Recipients were infected either with WT mCMV expressing all three vRAPs (indicted by red-filled capsid in the virus symbol) or with the triple deletion mutant mCMV-ΔvRAP lacking all three vRAPs (indicated by red-rimmed capsid in the virus symbol). **B** Corresponding survival rates of the infected allo-HCT recipients. Graphically modified based on data from reference [52]

It is proposed that transplantation tolerance toward histocompatibility antigens is accompanied by a bystander non-cognate tolerance against viral antigens (discussed in [36]). Notably, enhanced antigen presentation by deletion of the vRAP-encoding genes in the infecting virus allowed survival under otherwise identical conditions of allo-HCTs (Fig. 5, [52, 53]). This finding excluded GvH-reactive cells as the cause of death, and instead revealed a critical role for sufficient antigen presentation to recruit also non-tolerized low-avidity CD8 T cells to antiviral protection.

## Synopsis

**Fig. 6:**
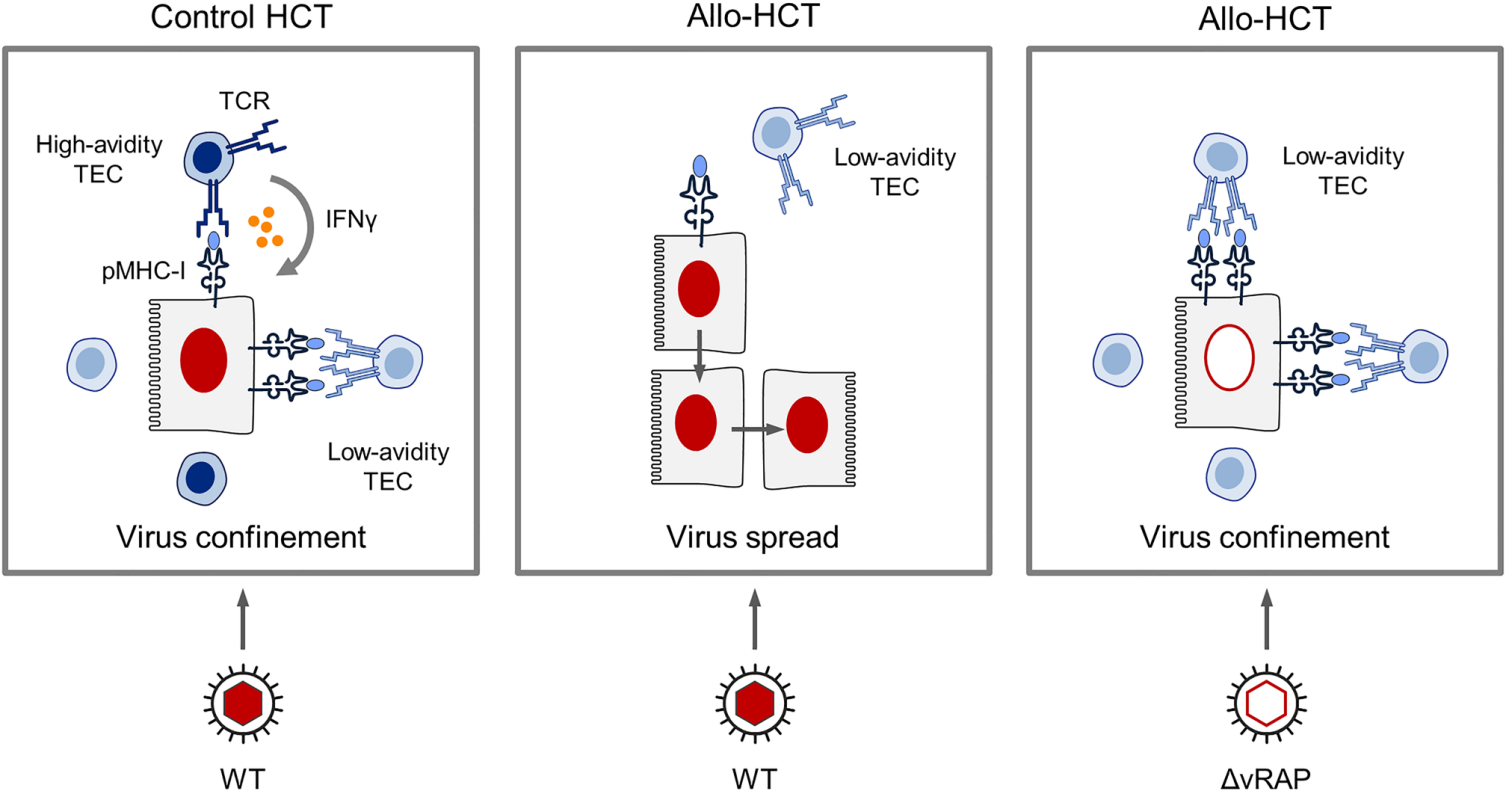
Graphical abstract. Symbols in light and dark blue color indicate low avidity and high avidity, respectively. *TEC* T effector cell. The filled red nucleus indicates the infection of tissue cells with wild-type (WT) mCMV expressing vRAPs. The red-rimmed empty nucleus indicates infection of tissue cells with the vRAP deletion mutant mCMV-ΔvRAP. See the body of the text for more detailed explanation. Reproduced from reference [36] in a graphically modified and rearranged form

We have here reviewed recent data from murine models of CD8 T-cell immunotherapy and allo-HCT that demonstrate the importance of CD8 T-cell avidity for controlling CMV, and that link the requirement of high-avidity to the limitation of antigen presentation by the concerted function of vRAPs. A most instructive example is lethal CMV disease associated with allo-HCT (Fig. 6, Graphical Abstract).

In control HCTs, designed to avoid a recognition of histocompatibility antigens in the recipient, high-avidity TEC are reconstituted and recognize infected cells despite the action of immune evasive vRAPs. They confine and eventually clear the infection by tissue infiltration and accumulation in microanatomical structures, known as “nodular inflammatory foci” (NIF) (see Fig. 4C), which can serve as histological indicators of protection. Importantly, IFNγ secreted by sensitized high-avidity TEC enhances antigen presentation [54] and thereby recruits also low-avidity TEC into protective NIF (Fig. 6, left panel). In contrast, in allo-HCT, only low-avidity TEC are reconstituted, which fail to become sensitized through interaction with a too low number of presented pMHC-I complexes. As a consequence, infected cells are not recognized and TEC are not recruited into NIF, so that the virus spreads uncontrolled and leads to a lethal histopathology (Fig. 6, center panel). Finally, deletion of vRAPs in the infecting virus prevents the inhibition of antigen presentation, so that even low-avidity TEC reconstituted by allo-HCT become sensitized by TCR-pMHC-I clustering, infiltrate infected tissue, and control the infection within NIF (Fig. 6, right panel).

In summary, all data provide strong evidence to conclude that immune evasion proteins do not prevent but only limit the cell surface display of pMHC-I complexes. They thereby raise the TEC avidity threshold required for the recognition of infected cells and thus for protection against uncontrolled virus spread, viral histopathology, and CMV organ disease. This new understanding solves the long-standing conflict between “immune evasion” of CMVs and the undisputable medical fact that CMV disease is a typical “disease of the immunocompromised host”.
